# Multicomponent intervention versus usual care for management of hypertension in rural Bangladesh, Pakistan and Sri Lanka: study protocol for a cluster randomized controlled trial

**DOI:** 10.1186/s13063-017-2018-0

**Published:** 2017-06-12

**Authors:** Tazeen H. Jafar, Imtiaz Jehan, H. Asita de Silva, Aliya Naheed, Mihir Gandhi, Pryseley Assam, Eric A. Finkelstein, Helena Legido Quigley, Marcel Bilger, Aamir Hameed Khan, John David Clemens, Shah Ebrahim, Elizabeth L. Turner, for COBRA-BPS Study Group, Anuradhani Kasturiratne

**Affiliations:** 1 Program in Health Services & Systems Research, Duke-NUS Medical School, 8 College Road, Singapore, 169857 Singapore; 2 Duke Global Health Institute, Duke University, Durham, NC USA; 3 Department of Community Health Sciences, Aga Khan University, Karachi, Pakistan; 4 Clinical Trials Unit, Department of Pharmacology, Faculty of Medicine, University of Kelaniya, Ragama, Sri Lanka; 5 International Centre for Diarrhoeal Disease Research, Dhaka, Bangladesh; 6 Biostatistics, Singapore Clinical Research Institute, Singapore, 138669 Singapore; 7 Centre for Quantitative Medicine, Duke-NUS Medical School, Singapore, 16957 Singapore; 8 Saw Swee Hock School of Public Health, National University of Singapore, Singapore, 117549 Singapore; 9 Section of Cardiology, Aga Khan University, Karachi, Pakistan; 10 London School of Hygiene & Tropical Medicine, London, UK; 11 Department of Biostatistics and Bioinformatics, Duke University, Durham, NC USA

**Keywords:** Hypertension, Lifestyle, Home health, Behaviour change, Cardiovascular risk, Public health, Cluster RCT

## Abstract

**Background:**

High blood pressure (BP) is the leading attributable risk for cardiovascular disease (CVD). In rural South Asia, hypertension continues to be a significant public health issue with sub-optimal BP control rates. The goal of the trial is to compare a multicomponent intervention (MCI) to usual care to evaluate the effectiveness and cost-effectiveness of the MCI for lowering BP among adults with hypertension in rural communities in Bangladesh, Pakistan and Sri Lanka.

**Methods/design:**

This study is a stratified, cluster randomized controlled trial with a qualitative component for evaluation of processes and stakeholder feedback. The MCI has five components: (1) home health education by government community health workers (CHWs), (2) BP monitoring and stepped-up referral to a trained general practitioner using a checklist, (3) training public and private providers in management of hypertension and using a checklist, (4) designating hypertension triage counter and hypertension care coordinators in government clinics and (5) a financing model to compensate for additional health services and provide subsidies to low income individuals with poorly controlled hypertension. Usual care will comprise existing services in the community without any additional training.

The trial will be conducted on 2550 individuals aged ≥40 years with hypertension (with systolic BP ≥140 mm Hg or diastolic BP ≥90 mm Hg, based on the mean of the last two of three measurements from two separate days, or on antihypertensive therapy) in 30 rural communities in Bangladesh, Pakistan and Sri Lanka. The primary outcome is change in systolic BP from baseline to follow-up at 24 months post-randomization. The incremental cost of MCI per CVD disability-adjusted life years averted will be computed.

Stakeholders including policy makers, provincial- and district-level coordinators of relevant programmes, physicians, CHWs, key community leaders, hypertensive individuals and family members in the identified clusters will be interviewed.

**Discussion:**

The study will provide evidence of the effectiveness and cost-effectiveness of MCI strategies for BP control compared to usual care in the rural public health infrastructure in South Asian countries. If shown to be successful, MCI may be a long-term sustainable strategy for tackling the rising rates of CVD in low resourced countries.

**Trial registration:**

ClinicalTrials.gov, NCT02657746. Registered on 14 January 2016.

**Electronic supplementary material:**

The online version of this article (doi:10.1186/s13063-017-2018-0) contains supplementary material, which is available to authorized users.

## Background

Cardiovascular disease (CVD) is a leading cause of mortality globally, accounting for 30% of deaths, even in low- and middle-income countries [1, 2]. In South Asia, high rates of CVD are observed at a younger age than in other countries, causing a greater loss of productive life years with severe economic consequences. Population blood pressure (BP) levels and rates of hypertension continue to rise in South Asia [3].

We have previously demonstrated the benefit of a model of care (COBRA: Control of Blood Pressure and Risk Attenuation), which combines family-based home health education (HHE) with trained private general practitioners (GPs), on lowering BP in urban Pakistan [4]. The combination of two interventions was superior than single or no intervention (usual care). However, the strategies were not evaluated in rural areas of Pakistan or any other South Asian country. Moreover, the urban model did not use the public health infrastructure for care delivery. Most of South Asia is still rural, where the prevalence of hypertension is high (one in four adults suffers from hypertension, and most cases are poorly controlled), and cardiovascular case fatality rates have been shown to be higher than in urban areas. Thus, evidence-based interventions applicable to rural South Asia are urgently needed.

Recently, we reported findings from a feasibility study to modify the COBRA model of care as a multicomponent intervention (MCI) for optimal delivery using the public health infrastructure in rural regions in Bangladesh, Pakistan and Sri Lanka (the COBRA-BPS feasibility study) [5]. The feasibility study also explored system-level barriers and facilitators of implementation of the MCI. Common standardized protocols and manuals for training community health workers (CHWs) and GPs in all three countries were developed. The feedback and suggestions from the stakeholders have already been incorporated in the full-scale trial protocol to be evaluated for fidelity, effectiveness and cost-effectiveness of the MCI model of care that we refined based on the COBRA-BPS feasibility study. The current protocol paper describes the full-scale COBRA-BPS trial in detail.

## Methods/design

We will evaluate the effectiveness of the MCI model of care compared to usual care in lowering systolic blood pressure (SBP) levels over 2 years among 2550 individuals aged 40 years or older with hypertension (those with SBP ≥140 mm Hg or diastolic blood pressure (DBP) ≥90 mm Hg based on the mean of the last two of three measurements from two separate days, or on antihypertensive therapy) in 30 rural communities in Bangladesh, Pakistan and Sri Lanka.

The MCI has five components: (1) HHE by government CHWs, (2) BP monitoring and stepped-up referral to a trained GP using a checklist, (3) training public and private providers in management of hypertension and using a checklist, (4) designating hypertension triage counter and hypertension care coordinators in government clinics and (5) a financing model to compensate for additional health services and provide subsidies to low income individuals with poorly controlled hypertension.

### Main and additional aims of the trial

The main aims of the trial are:

1. To evaluate whether the MCI (described with components 1–5 above) is better than usual care in lowering BP among adults with hypertension in rural communities
2. To quantify the incremental cost-effectiveness of the MCI approach in terms of cost per projected cardiovascular disease disability-adjusted life years (CVD DALYs) averted from the societal, government and participant perspectives.

Additional aims are:

3. To determine whether MCI improves medication adherence and lowers body mass index (BMI), dietary sodium, smoking rates, lipid levels, glucose levels and INTERHEART CVD risk score relative to usual care [6]
4. To determine whether MCI will lead to a greater reduction in 24-h ambulatory blood pressure among individuals with poorly controlled hypertension (defined as those with persistently elevated SBP >140 mm Hg or DBP >90 mm Hg mean of last two of three measurements from two separate days and receiving antihypertensive medications) in rural communities in Bangladesh, Pakistan and Sri Lanka

### Qualitative aims

A complementary qualitative study is planned in the MCI arm with the following objectives: (1) to understand facilitators and barriers to MCI from a stakeholder perspective and (2) to seek the hypertensive individuals’ experience during the course of MCI strategy in relation to components of the intervention, diagnosis of hypertension, treatment, quality of life and sustainability of intervention through in-depth interviews.

The central hypotheses of the study are as follows:

1. MCI will be more effective relative to usual care in lowering the BP of adults with hypertension in rural communities in Bangladesh, Pakistan and Sri Lanka.
2. MCI will be more cost-effective relative to usual care when measured against common thresholds for cost-effectiveness.

### Trial design

**Fig. 1:**
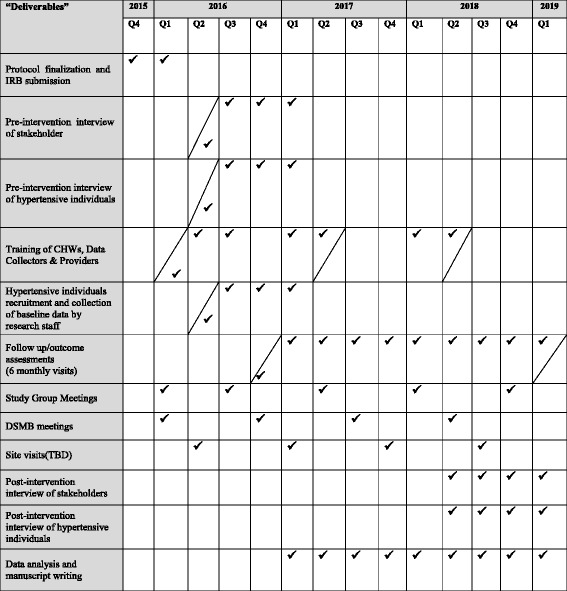
SPIRIT figure

**Fig. 2:**
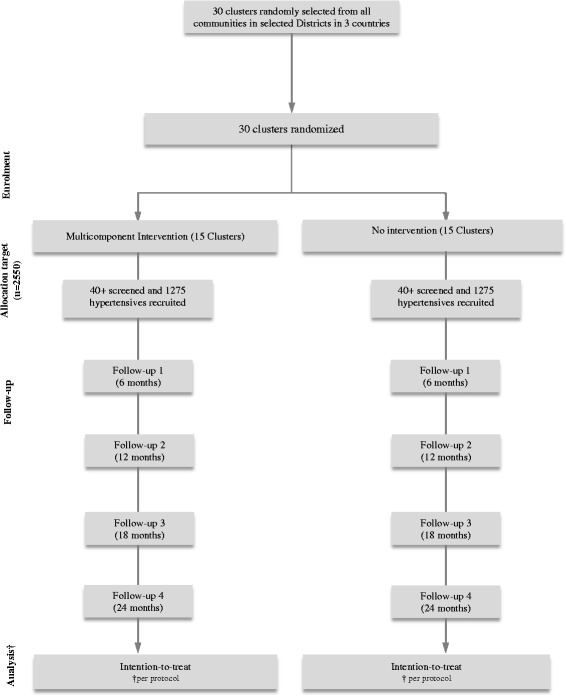
Study flow diagram. † Participants who successfully completed 2 years of follow-up will be included in the analyses

The study is a stratified cluster randomized controlled trial (RCT) in rural communities in three South Asian countries conducted according to the UK Medical Research Council’s (MRC’s) framework for implementing complex intervention trials [5]. A cohort of 850 individuals with hypertension will be recruited in each country and followed over a 2-year period in order to be able to evaluate the BP change within individuals. Ethical approval has been obtained from the Ethics Review Committees of the International Centre for Diarrhoeal Disease Research, Bangladesh (ICDDR, B) Aga Khan University, the University of Kelaniya, the London School of Hygiene & Tropical Medicine (LSHTM) and Duke-NUS Singapore. A Standard Protocol Items: Recommendations for Interventional Trials (SPIRIT) checklist is provided as Additional file 1. The SPIRIT deliverable items are shown in Fig. 1. The flow diagram for the study protocol is included as Fig. 2.

### Trial setting and randomization

The trial will be conducted in Bangladesh, Tangail District (population 3.2 million) and Munshiganj District (population 1.4 million); Pakistan, Thatta District (population 1.5 million); and Sri Lanka, Puttalam District (population 1.6 million). The unit of randomization will be a cluster defined by 250–300 households as defined by local administration according to CHW catchment area (each served by one to two CHWs). These clusters are grouped in geographically contiguous administrative units (AUs) as defined by the local governments (12 sub-districts in Tangail, 6 sub-districts in Munshignaj, 30 union councils in Thatta, 12 medical officers of the health division in Puttalam District) such that each unit is served by one government clinic. First, in the selected district of each country, 10 administrative units will be deliberately sampled, and the respective government clinic will be determined. Within each AU in each country, an eligible cluster will be identified (one cluster is defined as a village for Bangladesh, two to five neighbouring villages for Pakistan and two Grama Niladhari (GN) divisions for Sri Lanka). Each country will measure the distance of clusters from the respective government clinic measured by a GPS device. In each AU, clusters will be stratified by their distance to respective government clinic into two strata: FAR and NEAR (a distance of 2 km or less will be defined as NEAR and more than 2 km as FAR). In each arm (usual care or MCI), five AUs will be further randomized into three NEAR and two FAR.

One NEAR cluster from each NEAR AU and one FAR cluster from each FAR AU will be randomly selected for subject recruitment. A minimum distance of 10 km between randomized clusters will be ensured. Randomization will be stratified by country as well as by the distance from the government clinic, and AUs will be randomized in a 1:1 ratio to either MCI or usual care within the six strata defined by the combination of country and distance (NEAR versus FAR) using a computer-generated randomization program at Singapore Clinical Research Institute, Singapore.

### Eligibility and recruitment

Inclusion criteria include individuals who:

1. Are of age ≥40 years
2. Reside in the selected clusters
3. Have hypertension defined either as:
  - Persistently elevated BP (SBP ≥140 mm Hg or DBP ≥90 mm Hg) from each set of last two of three readings from two separate days, where BP measurements on the same day were measured at least 3 min apart
  - Are maintained on antihypertensive medications
4. Are willing to give informed consent

Exclusion criteria include:

1. Permanently bedridden individuals too ill to commute to the clinic
2. Pregnant women or individuals with advanced medical disease (those on dialysis, with liver failure or with other systemic diseases)
3. Individuals who are mentally compromised and unable to give informed consent

### Intervention

The MCI will be rolled out in the 15 clusters assigned to the intervention in the three countries (five in each). Components of the MCI include:

1. *Home health education (HHE) by government CHWs:* The standardized curricula have been adapted to local diets and languages in the three countries during the pilot feasibility study [5]. Training for CHWs in conveying HHE using a structured behaviour change communication approach will be carried out by a qualified nutritionist for 3.5 h/day over 5 days (see details in Additional file 2). During this time the CHWs will also be trained in completing a paper-based HHE checklist developed as part of the MCI (Additional file 3). The health messages include information on the deleterious effects of hypertension, non-pharmacological interventions for preventing and controlling hypertension and CVD, along with advice on weight loss strategies, physical activity, smoking cessation, avoiding excessive alcohol, low salt and saturated fat intake, and high fruit and vegetable intake.
  The trained CHWs will go door to door and deliver the sessions every 3 months in each cluster (about 60 households with hypertension) per CHW — a caseload consistent with the existing services. CHWs will deliver HHE to all individuals with elevated BP (SBP ≥140 mm Hg or DBP ≥ 90 mm Hg) at any visit. The first HHE session is expected to run for one hour with the second session expected to last 30 minutes. Family members (including those without hypertension) are also invited to sit in the sessions. The HHE Checklist will be completed by the CHW at the end of the session and submitted to the Local Health Office (LHO) via the community health supervisor.
2. *BP monitoring, and stepped-up referral to trained GP using checklist:* Every government CHW in the 15 intervention clusters will have a digital portable BP device (Omron HEM-7300™ Blood Pressure Monitor) and will be rigorously trained in standardized methods for BP measurement. CHWs will monitor the BP of all individuals aged 40 years or older with hypertension (as defined at baseline) at 3-monthly intervals and will complete the CHW BP monitoring and HHE checklist at each visit (Additional file 3) including list of HHE sessions delivered. All individuals with poorly controlled BP (SBP ≥160 mm Hg or DBP ≥100 mm Hg) at any visit will be referred to a trained GP. The CHW will complete and submit the GP management checklist (Additional file 4) to the CHW supervisor to track the referrals.
3. *Training providers in BP monitoring, management of hypertension and using checklist:* As in the feasibility study, GPs and midlevel providers (nurses, pharmacists) in public and private clinics will be invited for training. All providers will be trained in standardized BP measurement. In addition, GPs will be trained in management of hypertension. The Hypertension Management Manual for Clinic Providers has been developed during the pilot feasibility study and includes training on (1) measurement of BP, (2) provision of lifestyle advice, (3) prescription of antihypertensive therapy and (4) risk stratification for lipid lowering. Those individuals with any of the following will be classified as being at ‘high risk for CVD’: (a) aged 55 years older with SBP ≥160 mm Hg (b) past history of diabetes, (c) past history of heart disease, (d) past history of stroke or (e) current smokers; these individuals will be initiated on statins. The GPs will also be trained in completing a GP referral checklist (Additional file 5). A pre- and post-test of knowledge and practices will be administered. As in the feasibility study, we anticipate at least 66% of all GPs in the intervention clusters to be trained.
4. *Designating hypertension triage counter and hypertension care coordinator at the government clinics:* A functional hypertension triage counter will be established at the government health clinic enrolled in the study. This triage counter will facilitate care of hypertensive individuals who present to the clinic with a GP referral checklist. The counter will be equipped with a digital BP device for standardized measurement of BP by a trained clinic nurse/assistant before evaluation by a trained physician. Additionally, a hypertension care coordinator will be appointed at each government clinic to facilitate tracking of referrals by the CHW of individuals with poorly controlled BP.
5. *Compensation for additional health services and targeted subsidies:* The government CHWs will receive a pre-decided compensation of up to 0.2 FTE equivalent upon submission of the CHW BP monitoring and HHE checklist. Hypertensive individuals who are deemed poor (according to local policy in each country developed by the study team) with poorly controlled BP (SBP ≥160 or DBP ≥100 mm Hg) will receive a travel voucher for a clinic visit, and a free supply of antihypertensive medications will be provided. However, each country will have the flexibility to conform the subsidies to fit within the existing models of disbursements used in their respective systems, ensuring appropriate vetting for means testing, logistics and accountability of disbursement policy and procedures.

#### Treatment algorithm

A standardized protocol has been developed and refined by study nephrologists, cardiologists, internists and family physicians using non-pharmacologic and pharmacologic interventions following National Institute for Health and Care Excellence (NICE) guidelines [7]. The antihypertensive agents include calcium channel blockers (CCBs), angiotensin-converting-enzyme (ACE) inhibitors (ACEIs) or angiotensin receptor blockers (ARBs) and thiazide-like diuretics. The target BP will be SBP <140 mm Hg and DBP <90 mm Hg for all participants. Individuals with hypertension classified as being at ‘high risk for CVD’ based on the risk stratification will be treated with statins. Details are provided in the Antihypertensive Medication Treatment Algorithm (Additional file 6). As in the pilot feasibility study, prescription of inexpensive, generic antihypertensive medicines and statins has been encouraged.

### Usual care

Usual care will comprise existing services in the community without any additional training. Services include government health worker home visits for preventive maternal and child health and family planning services. However, these do not include formal screening or monitoring of BP or lifestyle advice for the prevention of hypertension. The existing clinic GPs in the communities (public and private) cater to 50–70 hypertensive individuals/day with an average contact time of 5–10 min per individual (per the pilot feasibility study) [5]. Whereas these providers are accustomed to providing care for common childhood illnesses and infectious diseases (diarrhoea, fever, malaria) that have traditionally burdened developing countries, the systems are not well organized for treatment of non-communicable diseases (NCDs), including hypertension.

Thus, usual care services do not include formal screening or monitoring BP or lifestyle advice for either prevention or management of hypertension; however, these services are included in the multicomponent intervention (MCI) arm.

### Screening and baseline data collection

The research staff masked to randomization status will first obtain a list of all members living in the households in the selected clusters from the Local Health Office and will visit all adults aged 40 years and older. Informed consent will be obtained for participation in the study. Those who consent will be screened for eligibility into the study. Standardized BP will be measured three times by research staff with a calibrated automated device, the Omron HEM-7300™ Blood Pressure Monitor, with the individual in a sitting position [8–10]. Readings will be taken 3 min apart. Individuals with elevated BP (SBP ≥140 mm Hg or DBP ≥90 mm Hg) of the last two of three readings on the first visit will be visited again after 2 weeks for re-measurement of BP to confirm hypertension (the BP measurements as described above will be repeated f), while those already on antihypertensive medications will be recruited at the first visit.

An interviewer-administered questionnaire translated into the local languages (Bangla, Urdu, Sindhi, Sinhala and Tamil) will be presented. Domains include socio-demographics, diet, lifestyle and behaviour, co-morbidities and health-related quality of life (EQ-5D-5L), health-seeking behaviours and associated costs. Adherence to antihypertensives and statins will be assessed by administering the Morisky scale [11]. Adiposity measures will be recorded. Fasting blood samples will be collected for plasma glucose, serum creatinine and lipids, and a morning spot urine sample for sodium, albumin and creatinine [12, 13].

In each of the 30 clusters, participants on antihypertensive medications with uncontrolled hypertension (defined as persistently elevated SBP >140 mm Hg or DBP >90 mm Hg from each set of two readings from 2 separate days) will be offered informed consent to undergo 24-h ambulatory BP monitoring (ABPM) with the Ultralight 90207™ model. Subjects meeting the eligibility criteria will be recruited consecutively (14 per cluster).

The MCI will be rolled out in the 15 clusters assigned to the intervention in three countries (five clusters in each). The CHW will then visit the household for HHE and will refer the individuals using the assigned GP referral checklist. The MCI team (including trainers and oversight personnel) and data collectors will be separate teams in each country. Although maintaining blinding at an operational level is challenging given the pragmatic nature of the MCI, measures will be taken to maintain masking of data collectors to randomization status by avoiding overlapping responsibilities of research data collectors and service delivery teams in the health care sector.

### Outcome measurements

Outcome assessors masked to randomization status will pay 6-monthly home visits for a total of 2 years. BP will be measured with the calibrated automated device Omron HEM-7300™ Blood Pressure Monitor (as in the baseline) with the hypertensive individual in the sitting position. Readings will be taken 3 min apart. The average of the last two of three readings will be considered in the analysis.

Information on behavioural risk factors (diet, physical activity, tobacco use and adiposity measures) will be obtained via an interviewer-administered questionnaire. Individuals will be asked to keep a record of the number of visits to the providers, type of facility (public or private), transportation costs, receipts of purchase of drugs and hospital visits. Based on our previous experience in urban Pakistan and the feasibility study in rural communities, the subjects are likely to maintain the records when requested in advance. Moreover, local information on market rates will be collected to supplement any missing information [5, 14]. Information on serious adverse events including death, hospitalization and any other serious events will be recorded.

The 24-h ABPM will be repeated at year 1 and at the final visit at year 2 post-randomization on the cohort enrolled in the ancillary study.

#### Primary effectiveness measure

The primary effectiveness measure is change in SBP from baseline to final follow-up at 2 years post-randomization. Both measurements will be taken by independent data collectors who will be masked to randomization.

#### Secondary effectiveness measures

The secondary effectiveness measures are as follows: (1) BP controlled to target (SBP <140 mm Hg and DBP <90 mm Hg; (2) composite outcome of death (all cause) or hospital admission due to coronary heart disease (CHD), heart failure or stroke; (3) incremental cost per quality-adjusted life year (QALY) gained from baseline to end of follow-up [15–17]; (4) change in antihypertensive medication adherence; (5) change in BMI; (6) change in dietary salt intake (urinary excretion); (7) change in prevalence of current smokers; (8) incident diabetes; (9) change in serum lipid levels; (10) change in INTERHEART CVD risk score; (11) incidence of adverse outcomes (medication side effects, sick days absenteeism, low QALY between randomized groups); (12) change in estimated glomerular filtration rate (eGFR); (13) change in urine albumin excretion.

#### Primary cost-effectiveness measures

The primary cost-effectiveness measures are incremental cost per mm Hg BP reduction from baseline to end of follow-up at 2 years post-randomization and incremental cost per projected CVD disability-adjusted life year (DALY) averted [14, 18].

#### Secondary cost-effectiveness measure

The secondary cost-effectiveness measure is incremental cost per QALY gained from baseline to end of follow-up [15–17].

#### Ancillary study outcome

The primary outcome for this sub-study is change in 24-h ambulatory mean SBP from baseline to final follow-up. The secondary outcome is 24-h ambulatory mean DBP.

##### Process measures

Fidelity and quality of implementation will be assessed, and explanations for potential discrepancies in observed and expected outcomes will be sought, which will aid future scale-up. As in the pilot study [5], these measures will include:

1. Proportion of planned HHE sessions delivered at the household level (HHE session checklist collected from CHW)
2. Proportion of individuals with hypertension referred by CHW to trained physician (physician referral checklist collected from hypertensive individuals)
3. Proportion of individuals with hypertension evaluated by trained physician (physician management checklists collected from the district health office)

In addition, deviations from the MCI protocol and also participant and health care provider perceptions of the quality and implementation of the intervention will be obtained.

### Statistical analysis

All main analyses will be based on the intention-to-treat population so that individuals will be analysed in the arm to which they were randomized, even if they did not adhere to the randomization (e.g. if an individual in the MCI arm did not participate in the MCI intervention). The chief principal investigator (PI) and site PIs will be masked to randomization status of all analytic data. The primary outcome is SBP change from baseline to 2 years. The four 6-monthly changes from baseline measurements (at 6, 12, 18 and 24 months) for all subjects will be modelled simultaneously using a likelihood-based generalized linear mixed model for repeated measures (MMRM) approach. This approach is a subject-level analysis that incorporates a cluster random effect. Although a Gaussian distribution with identity link function is pre-specified for the primary outcome, appropriate distributions within the exponential family and corresponding link functions will be employed in the case of non-normality. An unstructured matrix will be used to model the within-subject variance-covariance structure. If this model fails to converge, other variance-covariance structures will be considered. Primary analyses models will include fixed effects for baseline SBP, country, indicator for distance from clinic (FAR or NEAR), age, gender, intervention arm, visit number and the intervention arm-by-visit number interaction. The primary outcome of interest at 2 years post-randomization will be estimated with corresponding 95% confidence interval using the appropriate contrast at the final visit. Additional analyses will include interactions for country and treatment group to determine whether the effects differ by country. If the interaction effect is found to be clinically meaningful, an MMRM model similar to the primary analysis model will be performed separately for each country. Other analyses (sensitivity, secondary and subgroup analyses) will be explained in a detailed statistical analysis plan paper.

### Sample size

The planned sample is 2550 subjects with hypertension, involving 3 strata (countries), with an equal number of clusters per strata (10 clusters per country or 30 clusters total) and similar cluster sizes (85 hypertensive subjects per cluster). Based on findings observed in a previous study in urban Pakistan, and assuming a conservative intraclass correlation coefficient (ICC) of 0.02, 80% follow-up within clusters at 2 years post-randomization (68 hypertensive subjects per cluster) and a two-sided type I error rate of 5%, the trial will provide >99% power for the overall test to detect a difference between arms in SBP reduction as small as 4 (SD 11) mm Hg [4, 14, 18]. The study will use 5 mm Hg as the clinically meaningful difference between the two arms for reduction in SBP.

If heterogeneity in intervention effect across countries is evident (e.g. a reduction in SBP of 3 (SD 11) mm Hg in one country and 9 (SD 16) mm Hg in the other two countries), the trial has 80% power to detect such differences in intervention effects based on the following assumptions: ICC of 0.02 and type I error rate of 1.6% (based on a Bonferroni adjustment), 30 clusters and 85 participants per cluster. In addition to this, the trial has >80% power to detect a difference of 4 mm Hg (SD 11) in SBP reduction between the MCI and usual care arms for each country separately, for an ICC of 0.02, a type I error rate of 5% and 10 clusters of size 85 subjects per country. Furthermore, the high power also ensures that the main effect is adequately powered even after adjusting for dropouts, at both the participant and cluster levels. Therefore, the study is not over-powered for heterogeneity or dropouts (missing data). Power and Sample Size (PASS) software was used for the power calculations. Based on our previous work in urban Pakistan, and expecting a somewhat lower attrition rate in rural areas, the attrition rate is likely to be less than 15% at the end of 2 years in the overall study; therefore, our assumptions about follow-up rates are conservative [4].

### Data monitoring

The chief PI (THJ) will be responsible for the overall management of the trial. The day-to-day management at each site will be the responsibility of the site PIs (AN, IJ, AdS). The study management group will comprise the chief PI, statisticians (MG and PA) and validation statistician (ELT), health economists (EAF and MB), qualitative expert (HLQ), site PIs and co-PIs, data managers, project coordinators and research assistants. The Trial Steering Committee (TSC) comprises an independent chair (a senior professor of cardiovascular disease) plus independent experts in statistics, qualitative and mixed-methods research, chronic disease advocacy and health policy and finance. An independent Data Safety and Monitoring Board (DSMB) has been established as per MRC guidelines to review quality and safety issues. The TSC and DSMB operate in line with the MRC terms of reference as amended and agreed on by members at their first meeting.

### Qualitative study: facilitators and barriers to multicomponent public health intervention

#### Conceptual framework

The aim of this study is to identify patient and health care provider experience relating to hypertension awareness, treatment and management; this study will be guided by the behaviour change theoretical framework. This framework draws on theories from implementation research [19] and behaviour change [20]. It encapsulates the barriers to hypertension control and makes it possible to explore mediating pathways and moderators [21]. We will explore sub-themes for investigating the implementation of evidence-based practice, organized under three main themes whereby a change in behaviour requires a strong commitment for change (intention barriers), the necessary skills and abilities to perform the behaviour (capability barriers), with no health system constraints, as well as barriers that are external to patients’ or health care providers’ control (health care system barriers) [22].

The second stage of this study will involve conducting semi-structured interviews with hypertensive individuals, CHWs, health care professionals, providers and policy makers. These interviews will be conducted annually at three time points: prior to the intervention, after 1 year of intervention and after the study completion. The study will adopt a thematic analysis of semi-structured interviews using an adaptation of a behaviour change theoretical framework and a health system’s assessment framework, adopting some techniques of grounded theory such as line-by-line analysis, identifying deviant cases and using the constant comparison method (Table 1).

**Table 1:** Number of participants per country over a 2-year period

Time points Participants	Prior to intervention	After 1 year of the intervention	After 2 years of the intervention
Hypertensive patients	20	20	20
Health care professionals	15	–	15
Policy makers	7–10	–	7–10
Total	42–45	20	42–45

The interviews will be audio recorded and translated directly from the respective languages to English on transcripts by an expert bilingual, native speaker. Deductive and inductive data analyses will be used. Relevant quotes representative of the analysis will be incorporated into the publications. Data will be managed and analysed using QSR International’s NVivo 10™ software.

### Economic evaluation

Both a budgetary impact analysis and a cost-effectiveness analysis will be performed, the latter using the intention-to-treat principle. The budgetary impact analysis will track the total and per participant costs (including travel time costs monetized at local wage rates) of program delivery using an activity-based costing (ABC) approach. Using this approach, all relevant labor, materials and supplies, contracted services, travel vouchers and opportunity costs required to deliver the interventions will be captured by key activities. This information will not only feed into the cost-effectiveness analysis, but it will also allow policy makers within each country to identify the full and public sector costs of the program, should it be expanded beyond the trial communities.

The incremental cost-effectiveness evaluation will follow the approach we have employed in prior studies [23] and focus on the additional cost per unit reduction in CVD DALYs relative to standard care. The DALY is a generic measure of the burden of disease that combines healthy life years lost because of premature mortality with those lost due to disability. The metric thus enables assessment of burden of disease and the extent of burden that can be removed due to successful interventions. Using this approach will allow for comparing our results with other programs aimed at improving health outcomes and against common thresholds for cost-effectiveness, such as WHO-CHOICE recommendations [24].

CVD DALYs averted will be computed using the approach presented in Jafar et al., which assumes a linear relationship between blood pressure and DALYs such that a 1 mm Hg reduction in BP leads to a 2.2% reduction in CVD DALYs [14, 18].

In addition, we will quantify incremental costs per QALY gained over the 2-year time period based on the EQ-5D-5L scores. This analysis assumes that there may be benefits to participants irrespective of changes in blood pressure.

All analyses will be conducted from a societal perspective (including payer and participant costs and benefits) and separately from a third party payer’s perspective, as results from the latter may be most relevant to potential government payers. Results will be estimated for the three countries together for wider generalizability to South Asia and other low and/or middle income countries (LMICs), and for each country separately for local applicability, and will be compared to established benchmarks for cost-effectiveness [25]. One-way and *n*-way sensitivity analyses and cost-effectiveness acceptability curves that graphically present the probability that the MCI is cost-effective for a range of willingness-to-pay metrics that a decision maker may consider will also be presented. Based on the results, we will discuss the extent to which the MCI is likely to be cost-effective in rural communities in other LMICs.

## Discussion

High BP is the leading attributable risk factor for disability and mortality from CVD globally, and it accounts for a third of age-standardized deaths in South Asia [26]. The early age of onset of CVD in South Asians worsens the consequences through a reduction in productive life years. About one in four adults suffers from hypertension in Bangladesh, Pakistan and Sri Lanka [27–29]. Evidence on effective health systems strategies to manage hypertension is limited, especially in rural South Asia where case fatality rates from CVD are even higher [30].

In the COBRA-BPS study, we will evaluate the effectiveness and cost-effectiveness of a comprehensive ‘multicomponent intervention (MCI)’ for effective delivery of hypertension care using the rural predominantly public primary care infrastructure. The MCI approach has been modified from a two-component strategy of HHE and training of GPs, previously shown to be successful in an urban private practice setting, into a five-component MCI with the additional components essential for monitoring of individuals with poorly controlled hypertension and sustainable care delivery via subsidies for travel to clinic and antihypertensive medications through the public health care infrastructure.

The MCI is also expected to strengthen the capacity of the health system by establishing a team-based approach and referral links with documentation of standardized care to enhance hypertension management. The trial uses hypertension as an entry point into developing rural NCD health services, and the HHE component of our intervention is the first-line approach for management of diabetes and other common NCDs. The use of statins among individuals at high risk for CVD follows an up-to-date comprehensive CVD risk reduction strategy. Models of coordinated care are recommended by the World Health Organization (WHO) for communicable disease programs, such as directly observed treatment (DOT) for tuberculosis, HIV and management of malaria [31]. Our trial will provide direct evidence of the value of using comparable models and platforms for NCD management. Furthermore, the training components in the intervention leverage the existing infrastructure and will create economies of scale during roll out at national and regional levels if the trial is successful. NCDs have recently been featured on the national health policy agenda in Bangladesh, Pakistan and Sri Lanka. Contrasting the experiences from these three countries should provide valuable lessons to implement an action plan and also validate the usefulness of our approach for other countries in the region and beyond.

## Trial status

Participants are currently being recruited. The first patient was enrolled in April 2016. The study SPIRIT timeline details are shown in Fig. 1.

## Additional files

Additional file 1: SPIRIT checklist. (PDF 136 kb)

Additional file 2: Summary of intervention trainings. (PDF 13 kb)

Additional file 3: CHW checklist. (PDF 175 kb)

Additional file 4: GP management checklist. (PDF 145 kb)

Additional file 5: GP referral checklist. (PDF 127 kb)

Additional file 6: Antihypertensive Medication Treatment Algorithm. (PDF 651 kb)

## Abbreviations

BPS: Bangladesh, Pakistan, Sri Lanka
CHW: Community health worker
COBRA: Control of Blood Pressure and Risk Attenuation
CVD: Cardiovascular disease
GP: General practitioner
HHE: Home health education
MCI: Multicomponent intervention
SPIRIT: Standard Protocol Items: Recommendations for Interventional Trials

## References

[1] Lopez AD, Mathers CD, Ezzati M, Jamison DT, Murray CJ (2006). Global and regional burden of disease and risk factors, 2001: systematic analysis of population health data. Lancet.

[2] Lozano R, Naghavi M, Foreman K, Lim S, Shibuya K, Aboyans V, Abraham J, Adair T, Aggarwal R, Ahn SY (2012). Global and regional mortality from 235 causes of death for 20 age groups in 1990 and 2010: a systematic analysis for the Global Burden of Disease Study 2010. Lancet.

[3] Forouzanfar MH, Liu P, Roth GA, Ng M, Biryukov S, Marczak L, Alexander L, Estep K, Abate KH, Akinyemiju TF (2017). Global burden of hypertension and systolic blood pressure of at least 110 to 115 mm Hg, 1990-2015. JAMA.

[4] Jafar TH, Hatcher J, Poulter N, Islam M, Hashmi S, Qadri Z, Bux R, Khan A, Jafary FH, Hameed A (2009). Community-based interventions to promote blood pressure control in a developing country: a cluster randomized trial. Ann Intern Med.

[5] Jafar TH, de Silva A, Naheed A, Jehan I, Liang F, Assam PN, Legido-Quigley H, Finkelstein EA, Ebrahim S, Wickremasinghe R (2016). Control of blood pressure and risk attenuation: a public health intervention in rural Bangladesh, Pakistan, and Sri Lanka: feasibility trial results. J Hypertens.

[6] McGorrian C, Yusuf S, Islam S, Jung H, Rangarajan S, Avezum A, Prabhakaran D, Almahmeed W, Rumboldt Z, Budaj A (2011). Estimating modifiable coronary heart disease risk in multiple regions of the world: the INTERHEART Modifiable Risk Score. Eur Heart J.

[7] National Institute for Health and Care Excellence. Hypertension in adults: diagnosis and management. 2016. http://guidance.nice.org.uk/CG127/QuickRefGuide/pdf/English. Accessed 12 Feb 2017. 31577399

[8] Coleman A, Steel S, Freeman P, de Greeff A, Shennan A (2008). Validation of the Omron M7 (HEM-780-E) oscillometric blood pressure monitoring device according to the British Hypertension Society protocol. Blood Press Monit.

[9] Wijewardene K, Mohideen M, Mendis S, Fernando D, Kulathilaka T, Weerasekara D (2005). Prevalence of hypertension, diabetes and obesity: baseline findings of a population based survey in four provinces in Sri Lanka. Ceylon Med J.

[10] Jafar TH, Levey AS, Jafary FH, White F, Gul A, Rahbar MH, Khan AQ, Hattersley A, Schmid CH, Chaturvedi N (2003). Ethnic subgroup differences in hypertension in Pakistan. J Hypertens.

[11] Lee GK, Wang HH, Liu KQ, Cheung Y, Morisky DE, Wong MC (2013). Determinants of medication adherence to antihypertensive medications among a Chinese population using Morisky Medication Adherence Scale. PLoS One.

[12] Mente A, O’Donnell MJ, Rangarajan S, McQueen MJ, Poirier P, Wielgosz A, Morrison H, Li W, Wang X, Di C (2014). Association of urinary sodium and potassium excretion with blood pressure. N Engl J Med.

[13] Calder J, Schachter M, Sever P (1993). Ion channel involvement in the acute vascular effects of thiazide diuretics and related compounds. J Pharmacol Exp Ther.

[14] Jafar T, Islam M, Bux R, Poulter N, Hatcher J, Chaturvedi N (2011). Cost-effectiveness of community-based strategies for blood pressure control in a low-income developing country: findings from a cluster-randomized, factorial-controlled trial. Circulation.

[15] Wang H, Dwyer-Lindgren L, Lofgren KT, Rajaratnam JK, Marcus JR, Levin-Rector A, Levitz CE, Lopez AD, Murray CJ (2012). Age-specific and sex-specific mortality in 187 countries, 1970-2010: a systematic analysis for the Global Burden of Disease Study 2010. Lancet.

[16] Li JS, Li SY, Yu XQ, Xie Y, Wang MH, Li ZG, Zhang NZ, Shao SJ, Zhang YJ, Zhu L (2012). Bu-Fei Yi-Shen granule combined with acupoint sticking therapy in patients with stable chronic obstructive pulmonary disease: a randomized, double-blind, double-dummy, active-controlled, 4-center study. J Ethnopharmacol.

[17] Vos T, Flaxman AD, Naghavi M, Lozano R, Michaud C, Ezzati M, Shibuya K, Salomon JA, Abdalla S, Aboyans V (2012). Years lived with disability (YLDs) for 1160 sequelae of 289 diseases and injuries 1990-2010: a systematic analysis for the Global Burden of Disease Study 2010. Lancet.

[18] Jakicic JM, Tate DF, Lang W, Davis KK, Polzien K, Rickman AD, Erickson K, Neiberg RH, Finkelstein EA (2012). Effect of a stepped-care intervention approach on weight loss in adults: a randomized clinical trial. JAMA.

[19] Michie S, Johnston M, Abraham C, Lawton R, Parker D, Walker A, Psychological TG (2005). Making psychological theory useful for implementing evidence based practice: a consensus approach. Qual Saf Health Care.

[20] Fishbein M (2000). The role of theory in HIV prevention. AIDS Care.

[21] Davies P, Walker AE, Grimshaw JM (2010). A systematic review of the use of theory in the design of guideline dissemination and implementation strategies and interpretation of the results of rigorous evaluations. Implement Sci.

[22] World Health Organization (2000). The world health report 2000: health systems: improving performance.

[23] Faqah A, Jafar TH (2011). Control of blood pressure in chronic kidney disease: how low to go?. Nephron Clin Pract.

[24] World Health Organization. Cost effectiveness and strategic planning (WHO-CHOICE). 2017. http://www.who.int/choice/costs/CER_levels/en/. Accessed 17 May 2017.

[25] Baltussen RMPM, Adam T, Tan-Torres Edejer T, Hutubessy RCW, Acharya A, Evans DB, Murray CJL. Making Choices in Health: WHO Guide to Cost Effectiveness Analysis. Geneva:World Health Organization; 2003.

[26] Lim SS, Vos T, Flaxman AD, Danaei G, Shibuya K, Adair-Rohani H, Amann M, Anderson HR, Andrews KG, Aryee M (2012). A comparative risk assessment of burden of disease and injury attributable to 67 risk factors and risk factor clusters in 21 regions, 1990-2010: a systematic analysis for the Global Burden of Disease Study 2010. Lancet.

[27] Jafar TH, Jessani S, Jafary FH, Ishaq M, Orakzai R, Orakzai S, Levey AS, Chaturvedi N (2005). General practitioners’ approach to hypertension in urban Pakistan: disturbing trends in practice. Circulation.

[28] Islam AK, Majumder AA (2012). Hypertension in Bangladesh: a review. Indian Heart J.

[29] Katulanda P, Ranasinghe P, Jayawardena R, Constantine GR, Rezvi Sheriff MH, Matthews DR (2014). The prevalence, predictors and associations of hypertension in Sri Lanka: a cross-sectional population based national survey. Clin Exp Hypertens.

[30] Yusuf S, Rangarajan S, Teo K, Islam S, Li W, Liu L, Bo J, Lou Q, Lu F, Liu T (2014). Cardiovascular risk and events in 17 low-, middle-, and high-income countries. N Engl J Med.

[31] Conseil A, Mounier-Jack S, Coker R (2010). Integration of health systems and priority health interventions: a case study of the integration of HIV and TB control programmes into the general health system in Vietnam. Health Policy Plan.

