# Supplementary material for: Multicomponent intervention versus usual care for management of hypertension in rural Bangladesh, Pakistan and Sri Lanka: study protocol for a cluster randomized controlled trial
Source: Trials. 2017 Jun 12;18:272. doi: 10.1186/s13063-017-2018-0 (PMC5469065; doi:10.1186/s13063-017-2018-0)
Supplement: Supplementary file 2 — Summary of intervention trainings. (PDF 13 kb) [file 13063_2017_2018_MOESM2_ESM.pdf]

Additional file 2: Summary of intervention trainings

Healthcare Provider	Training Components	Duration	Trainer
**Community health workers (CHW)**	1. Home Health Education (HHE)<br>2. Blood pressure measurement and interpretation and stepped-up referral to GP included in training on;<br>3. CHW BP Monitoring and HHE Checklist<br>4. Referral Checklist<br>5. Standardized lifestyle messages for better control of blood pressure<br>6. Behaviour change communication strategies	5 days<br>1 day follow-up refresher session will be held after 2 months and then<br>***Annual re-training***	Nutritionist
**Research staff**	1. Proper method of measuring and interpreting blood pressure<br>2. Identification of urgent cases and standard procedure for referring to higher levels of care<br>3. Proper method of taking anthropometric measurements<br>4. Procedure for obtaining and recording informed consent<br>5. Interview and data collection techniques<br>6. Standardized techniques of editing data collection forms<br>7. Realize and record adverse events during the follow up visits and report to the site PIs	1 day<br>re-training will be during 4 hour session after 3 months and then<br>***Annual re-training***	Study coordinators
**Providers (nurses, pharmacists/ dispensers) in public, and GPs in public and private clinics)**	*Hypertension Management Manual for Clinic Providers consists of training on:*<br>1. BP measurement using digital Omron device<br>2. Lifestyle advice<br>3. Anti-hypertensive treatment algorithm per protocol<br>4. Assessing risk factors and co-morbidities and risk stratification, and initiation of lipid lowering therapy<br>5. Clarity on mechanism of physician oversight<br>6. When to refer individuals to the next level of care<br>7. Maintain GP Management Checklist	1 day<br>re-training will be during 4 hour session after 3 months and then<br>***Annual re-training***	Family Physician or Hypertension Specialist e.g. Cardiologist, Nephrologist, Internist
