# Supplementary material for: Multicomponent intervention versus usual care for management of hypertension in rural Bangladesh, Pakistan and Sri Lanka: study protocol for a cluster randomized controlled trial
Source: Trials. 2017 Jun 12;18:272. doi: 10.1186/s13063-017-2018-0 (PMC5469065; doi:10.1186/s13063-017-2018-0)
Supplement: Supplementary file 3 — CHW checklist. (PDF 175 kb) [file 13063_2017_2018_MOESM3_ESM.pdf]

Primary Care Strategies to Reduce High Blood Pressure: A Cluster Randomized Trial in rural Bangladesh, Pakistan and Sri Lanka

CHW BP MONITORING and HOME HEALTH EDUCATION CHECKLIST

1	Date of interview (dd/mm/yyyy)		8	Number Assigned by CHW	
2	Country		9	Participant's name	
3	Cluster number		10	Gender	
4	Structure number		11	Visit Type (e.g. BL, 3m, 6m, 9m, …)	
5	Community household number		12	**Study ID (if applicable):** _ _ - _ _ - _ _ _ _ - _ _	
6	Family number				
7	Name of CHW				

Instructions: Please tick (✓) the box that corresponds to your response, indicate text answers on allotted space where applicable.

**(1) A. BP measurement Preparation**:	
a. Did the participant smoke in the past 30 minutes?	☐ 1. Yes ☐ 2. No
b. Did the participant have any caffeinated drink (e.g. coffee, tea) in the past 30 minutes?	☐ 1. Yes ☐ 2. No
c. Has the participant rested for at least 5 minutes prior to BP measurement?	☐ 1. Yes ☐ 2. No
Proceed to measure BP only if responses to questions a, b are "No" and for question c is "Yes"	

(1) B. Cuff selection:

Using the **medium-sized cuff** estimate if the

- bladder covers ≥ 80% of arm circumference: ☐ 1. Yes ☐ 2. No

OR

- Cuff width ≥ 40% of arm circumference: ☐ 1. Yes ☐ 2. No

If either of above "Yes", proceed to measure BP.

If both of above "No", measure arm circumference: _______ cm

Size of cuff used:

☐ 1. Medium ☐ 2. Large

Arm Circumference:

**≤32 cm**	Medium Cuff
**>32 cm**	Large Cuff

*Signature of CHW:*_________________________

**(1) C. BP measurement**:		
Ensure 1st and 2nd reading taken at least 3 minutes apart.		
**BP Readings**	**1st Reading**	**2nd Reading**
Time of BP Reading (hh:mm):		
Systolic BP (SBP) (mm Hg):		
Diastolic BP (DBP) (mm Hg):		
PULSE/min:		
Are **both** BP readings equal to or more than **(systolic 160 mm Hg or diastolic 100 mm Hg)**? ☐ 1. Yes ☐ 2. No		

(2) Has the participant consulted with a clinic healthcare provider since the last CHW visit?

☐ 1. Yes→Advice to follow-up with a clinic provider for 6-8 weeks later

☐ 2. No→ Advice to follow-up with a clinic provider for initiation of treatment **AND** flag for CHW Supervisor visit

CHW Supervisor Details
Name: ____
Position: ____
Date Notified (dd/mm/yyyy): ____
Method of Notification(e.g. phone, letter, personally): ________________________

(3) Is the participant currently taking any anti-hypertensive medication? ☐ 1. Yes ☐ 2. No

If **YES**, write down the details of the medication?

Generic Name: __________________________ **Dosage:** ____________ mg/tab

Frequency: ☐ Once daily ☐ 2 times/day ☐ 3 times/day ☐ Other: specify____________

(4) HHE Session details

Date (dd/mm/yyyy): ____
Time Started (hh:mm): ___________________ Time Ended (hh:mm):____________________
Number of participants during HHE: _______________________________
Number of HHE Participants with elevated BP: _______________________

☐ 1. Advised on nutrition (↓Foods high on saturated fats, ↓Salt intake, ↑Fruit and vegetables)
☐ 2. Advised on maintaining ideal body weight
☐ 3. Advised on physical activity
☐ 4. Advised on smoking cessation
☐ 5. Advised on adherence to high blood pressure medications
☐ 6. Advised on stress management
☐ 8. Other: ____

(5) Any action taken during this home visit? ☐ 1. Yes ☐ 2. No (→ END)
If **YES**, what are they? (Tick ✓ all check boxes that apply)

☐ 1. Advised to proceed to referral hospital **(**If SBP **180 mm Hg or more**, DBP **120 mm Hg or more**, acute conditions: **chest pain, shortness of breath, or any other serious illness that the CHW deems as requiring urgent referral to a hospital (specify)):**

____

☐ 2. Provided information about the participant to the referral hospital (list details of referral):
Referral Hospital: ____
Name of Healthcare Provider (if contacted):_______________________________________
Designation of Healthcare Provider (if contacted):_________________________________
Manner Contacted: ☐ 1. Call ☐ 2. Personally ☐ 8. Others (specify)________________

☐ 3. Advised to consult/follow-up with a trained General Practitioner
[IN INTERVENTION CLUSTERS ONLY]
