# Supplementary material for: Multicomponent intervention versus usual care for management of hypertension in rural Bangladesh, Pakistan and Sri Lanka: study protocol for a cluster randomized controlled trial
Source: Trials. 2017 Jun 12;18:272. doi: 10.1186/s13063-017-2018-0 (PMC5469065; doi:10.1186/s13063-017-2018-0)
Supplement: Supplementary file 4 — GP management checklist. (PDF 145 kb) [file 13063_2017_2018_MOESM4_ESM.pdf]

Primary Care Strategies to Reduce High Blood Pressure: A Cluster Randomized Trial in rural Bangladesh, Pakistan and Sri Lanka

GP MANAGEMENT CHECKLIST

1	Date of diagnosis (dd/mm/yyyy)		6	Participant's name	
2	Date of birth (dd/mm/yyyy)		7	Community Household #	
	Age at last birthday		8	Name of provider	
3	Name of CHW		9	Designation of provider	
4	Blood pressure as indicated in the referral checklist (average of 2 readings)	SBP: ______mm Hg<br>DBP: _____mm Hg	10	Clinic Address	
5	Number Assigned by CHW		11	Visit Type (eg. BL, 3m, 6m, 9m, …)	
**Study ID (if any):** _ _ - _ _ - _ _ _ _ - _ _					

(1) Details of BP Reading at the current clinic visit:

**BP Readings**	**1st Reading**	**2nd Reading**
Time of BP Reading (hh:mm):		
Systolic BP (mm Hg):		
Diastolic BP (mm Hg):		
Pulse (beats per minute):		

Is the current **systolic BP below 140 mm Hg and diastolic BP below 90 mm Hg** (Treatment Target) for persons aged <80 years?

☐ 1. Yes

☐ 2. No → *manage hypertension as per treatment algorithm*

Signature of the GP: ____________

(2) Is the patient currently on an anti-hypertensive medication?

☐ 1. Yes→Indicate details below

☐ 2. No→ Item **#3**

**No.**	**Generic Name**	**Dosage**	**Frequency** (# of times taken per day)	**Medication Status**
**1**	______________________	______ mg/tab		☐ 1. Newly started<br>☐ 2. Dose titrated<br>☐ 3. Dose maintained
**2**	______________________	______ mg/tab		☐ 1. Newly started<br>☐ 2. Dose titrated<br>☐ 3. Dose maintained
**3**	______________________	______ mg/tab		☐ 1. Newly started<br>☐ 2. Dose titrated<br>☐ 3. Dose maintained

(3) Does the patient have any of the following high CVD risk factors? *(Tick all check boxes that apply)*

☐ 1. Age ≥ 55 years and SBP ≥ 160 mm Hg

☐ 2. Diabetes Mellitus

☐ 3. History of heart disease

☐ 4. History of stroke

☐ 5. Current smoker

Prescribe **statin** if any of the above factors is present.

Name of statin prescribed	Dose of statin prescribed

 Signature of the GP: ____________

(4) Any action taken during this follow-up clinic visit?

☐ 1. Yes
If **YES**, what are the action(s) taken? *(Tick all check boxes that apply)*

- ☐ Advice on lifestyle modification strengthened
- ☐ Advice on medication adherence strengthened
- ☐ Anti-hypertensive medication prescribed
- ☐ Statin prescribed
- ☐ Other: ____

☐ 2. No (→ END)
