# Supplementary material for: Multicomponent intervention versus usual care for management of hypertension in rural Bangladesh, Pakistan and Sri Lanka: study protocol for a cluster randomized controlled trial
Source: Trials. 2017 Jun 12;18:272. doi: 10.1186/s13063-017-2018-0 (PMC5469065; doi:10.1186/s13063-017-2018-0)
Supplement: Supplementary file 5 — GP referral checklist. (PDF 127 kb) [file 13063_2017_2018_MOESM5_ESM.pdf]

Primary Care Strategies to Reduce High Blood Pressure: A Cluster Randomized Trial in rural Bangladesh, Pakistan and Sri Lanka

GP REFERRAL CHECKLIST

1	Date of interview (dd/mm/yyyy)		6	Participant's name	
2	Date of birth (dd/mm/yyyy)		7	Community Household #	
	Age at last birthday		8	Visit Type (eg. BL, 3m, 6m, 9m, …)	
3	Number Assigned by CHW				
4	Name and Signature of CHW	Name:<br>Signature:			
5	Name of healthcare provider and clinic/hospital being referred to	Name of healthcare provider:<br>Clinic/hospital:			

Is the participant any of the following: (Tick ✓ all check boxes that apply)

☐ 1. This patient was found to have persistently elevated blood pressure (**systolic ≥ 160 mm Hg or diastolic ≥ 100 mm Hg**) for **2 separate readings.**

☐ 2. This patient is currently having shortness of breath, chest pain or other serious medical condition

☐ 3. Specify condition/symptoms:____

☐ 4. Enter BP readings below:

**BP Readings**	**1st Reading**	**2nd Reading**
Time of BP Reading (hh:mm):		
Systolic BP (SBP) (mm Hg):		
Diastolic BP (DBP) (mm Hg):		
PULSE/min:		

Signature of staff:________________________
