# Supplementary figures and images for: Multicomponent intervention versus usual care for management of hypertension in rural Bangladesh, Pakistan and Sri Lanka: study protocol for a cluster randomized controlled trial
Source: Trials. 2017 Jun 12;18:272. doi: 10.1186/s13063-017-2018-0 (PMC5469065; doi:10.1186/s13063-017-2018-0)

### **Additional file6-Antihypertensive Medication Treatment Algorithm**

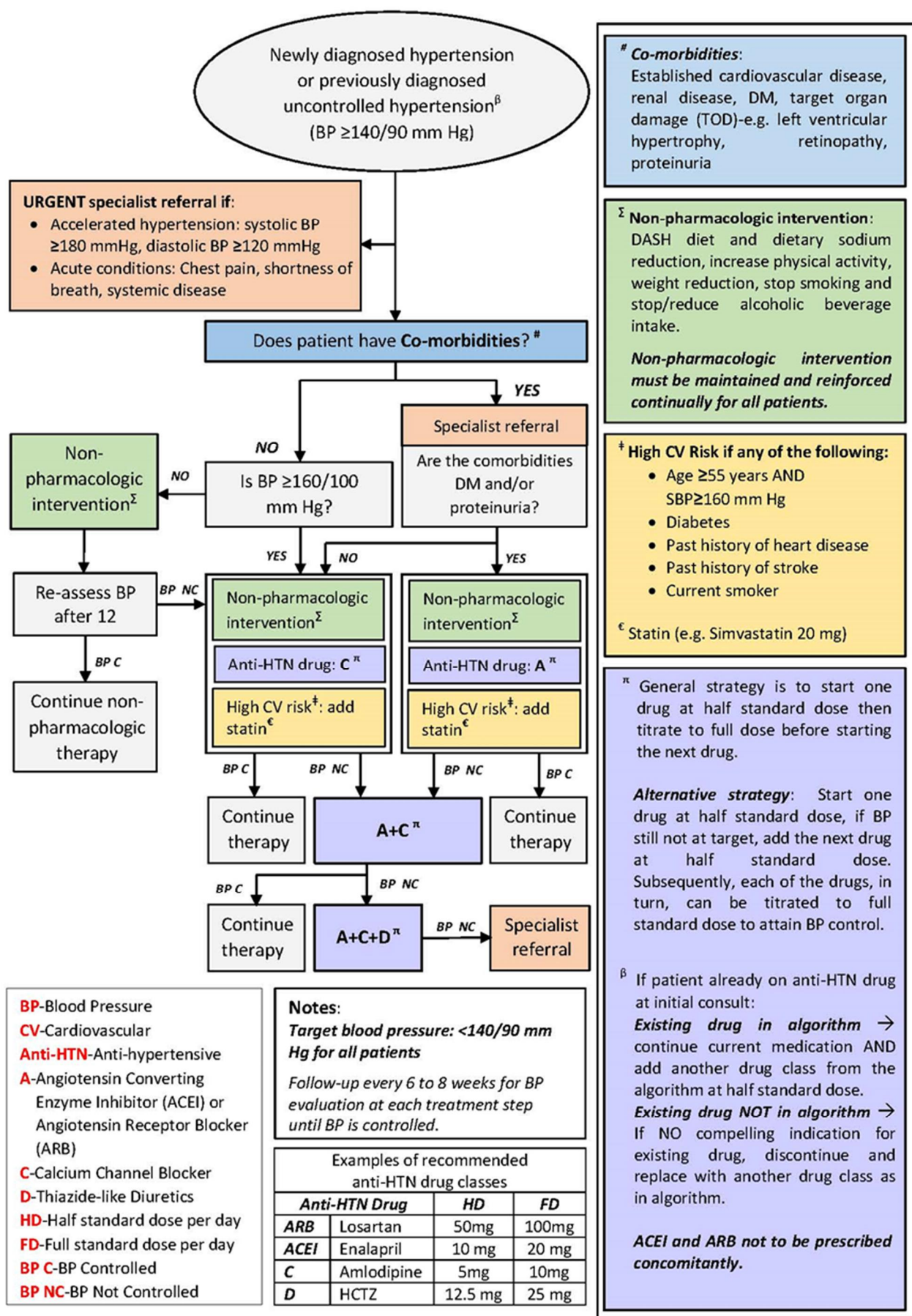

Supplement: Supplementary file 6 — Antihypertensive Medication Treatment Algorithm. (PDF 651 kb) [file 13063_2017_2018_MOESM6_ESM.pdf]
